# The effect of direct admission to acute geriatric units compared to admission after an emergency department visit on length of stay, postacute care transfers and ED return visits

**DOI:** 10.1186/s12877-022-03241-x

**Published:** 2022-07-04

**Authors:** D. Naouri, N. Pelletier-Fleury, N. Lapidus, Y. Yordanov

**Affiliations:** 1 Centre for Research in Epidemiology and Population Health, INSERM U1018), French National Institute of Health and Medical Research, Université Paris- Saclay, Université Paris-Sud, UVSQ, Villejuif, France; 2 Sorbonne Université, INSERM, Institut Pierre Louis d’Epidémiologie Et de Santé Publique IPLESP, AP-HP. Sorbonne Université, Saint-Antoine Hospital, Public Health Department, 75012 Paris, France; 3 UMR-S 1136, Sorbonne Université, APHP, Hôpital Saint Antoine, INSERM, Institut Pierre Louis d’Epidémiologie Et de Santé Publique, Service d’Accueil des Urgences, Paris, France

**Keywords:** Elderly, Emergency department, Emergency medicine, Geriatrics, Health Service Research

## Abstract

**Background:**

Compared with conventional hospitalization, admission to an acute geriatric care unit (AGU) is associated with better outcomes in elderly patients. In 2012, 50% of the hospitalizations of elderly patients were preceded by an emergency department (ED) visit. Hospital occupancy, access blocks and overcrowding experienced by patients during ED visits are associated with increased morbidity.

**Objective:**

Our aim was to evaluate the effect of direct admission (DA) to an AGU on both the hospital length of stay and morbidity of elderly patients.

**Design:**

This study was a retrospective cohort study conducted using electronic medical records and administrative claims data from the Greater Paris University Hospitals (APHP) health data warehouse involving 19 different AGUs.

**Participants:**

We included all patients ≥ 75 years old who were admitted to an AGU for more than 24 h between January 1, 2013, and December 31, 2018.

**Intervention:**

Direct admission to the AGU compared to admission after an ED visit.

**Main measures:**

The main outcome was hospital length of stay. Two outcomes were used to analyse morbidity: postacute care and rehabilitation ward transfer at the end of the index hospitalization and ED return visit within 30 days after the index hospitalization (for those who survived to hospitalization). We used an inverse probability of treatment weighting (IPTW) approach to balance the differences in patient baseline variables between the two groups. Univariate linear and logistic regression models were built to estimate the effect of DA on hospital length of stay and the likelihood of postacute care transfer and ED return visit.

**Key results:**

Among the 6583 patients included in the study, DA was associated with a lower hospital length of stay (estimate = -1.28; 95% CI = -1.76–0.80), and a lower likelihood of postacute care transfer (OR = 0.87; 95% CI = 0.77–0.97). It was not significantly associated with a lower risk of ED return visits (OR = 0.81; 95% CI = 0.60–1.08) in the following month.

**Conclusion:**

DA should be prioritized, and reorganization of the geriatric pathway around DA should be encouraged due to the frailty of elderly individuals.

**Supplementary Information:**

The online version contains supplementary material available at 10.1186/s12877-022-03241-x.

## Introduction

Elderly patients represent an increasing proportion of emergency department (ED) users [1]. In France in 2012, 50% of the hospitalizations of elderly patients were preceded by an ED visit [2]. Hospital occupancy, access blocks, ED waiting times and overcrowding have been described as being associated with a higher frequency of medical errors [3] and an increase in morbidity [4–10]during an ED visit for both hospitalized and discharged patients. This finding is even truer in elderly individuals, who are twice as likely to experience delirium related to an extended ED length of stay (LOS) [11] and to suffer from a 3% increase in the risk of adverse events per hour spent in the ED [12].

These findings encourage stakeholders to think about organizational changes and changes in professional practices [13, 14]. France is not the only country in Europe to face this situation. Several studies suggested that admission of elderly people to an acute geriatric care unit (AGU) compared with conventional hospitalization, was associated with better outcomes [15–17] less incident delirium [15], better functional status [16, 17] and a higher likelihood of returning home at hospital discharge [16, 17]. AGUs involve specialized multidisciplinary teams with direct responsibility not only for the care of elderly individuals with acute medical disorders but also for providing a geriatric assessment on care and care coordination. Several studies have shown that admission to an AGU is associated with better outcomes than admission to another hospital ward. In France, admission to an AGU is carried out in the neighbourhood hospital, i.e., depending on the place of residence. The care provided is generally homogeneous and standardized across the AGUs.

Some other studies assessed direct admission (DA) to an AGU [18, 19] compared to post-ED admission, but they showed contrasting results. The scarcity of studies on the topic and the low quality of the available evidence, due in particular to the monocentric nature of these studies, prevent us from drawing any conclusion on the possible benefits of either of these strategies [18–20].

In this study, we aimed to estimate the effect of DA to an AGU on both hospital LOS and morbidity in elderly patients compared with those admitted after an ED visit. We hypothesized that a DA strategy might be associated with both shorter hospital LOS and lower morbidity. The results of this study may contribute to the debate on the efficiency of care pathways for the elderly individuals.

## Methods

### Study design

This retrospective weighted-cohort study was conducted using electronic medical records and administrative claims data from the Greater Paris University Hospitals (Assistance Publique-Hôpitaux de Paris, APHP) health data warehouse [21]. APHP is a network of 39 university hospitals in the greater Paris area covering a large part of this area’s population (12 million inhabitants). These data include warehouse data from university hospitals on all patients treated in the APHP hospital network. The reporting of this study followed the Strengthening The Reporting of Observational Studies in Epidemiology (STROBE) guidelines [22].

### Setting

The APHP data warehouse contains medico-administrative and care data collected in the hospital information system, such as demographics, standardized hospitalization reports, the results from biological and radiological exams, diagnostic codes according to the 10th International Classification of Diseases (ICD-10 codes), diagnosis-related groups (DRG) and therapeutic interventions according to the French Common Classification of Medical Acts (Classification Commune des Actes Médicaux, CCAM). Our study involved data from 19 different AGUs of APHP university hospitals. This study was approved by the Scientific and Ethical Committee of APHP (IRB00011591).

### Study participants

We included all patients ≥ 75 years old who were admitted to an AGU for more than 24 h (inpatient care), between January 1, 2013 and December 31, 2018, with a complete file (hospitalization report and coding diagnosis). When patients were included several times in our cohort, we analysed their latest admission (index hospitalization). After taking into account the weightings from the propensity score (Inverse Probability Treatment Weighting (IPTW) propensity score, see below), patients admitted directly to the AGU were compared with patients admitted to the AGU after an ED visit (excluding those admitted via an intensive care unit or a non-geriatric specialty unit).

We excluded all patients with clinical signs of life-threatening conditions at ED presentation (presence of mottling, respiratory distress, cyanosis, indrawing and/or need for a fluid administration), reported in the ED medical records (identified via text mining) and those with medical conditions that did not respect the positivity assumption of propensity score (see below) [23]. The positivity assumption states that each subject has a nonzero probability of receiving either intervention, in this case DA to the AGU or admission via an ED visit. In our study, the positivity assumption of propensity score could not always be verified, especially for certain medical conditions that, due to their nature or mode of occurrence, required an ED visit before being admitted to the AGU. Details regarding text mining and diagnosis exclusions are available in Appendix 1.

### Study exposure and outcomes of interest

The studied exposure was patients’ DA to an AGU (DA group) as opposed to an admission after an ED visit (ED group). DA corresponded to an admission to AGU directly from the usual place of living (without ED referral), after clinical assessment, organized via telephone contact between the outpatient physician (mostof the time a general practitioner who knows the patient thus enabling better communication) and an AGU physician. The main outcome was hospital LOS, which was measured in all patients. For patients who died during their hospital stay, LOS was censored at the date of death.

Two outcomes were used to analyse morbidity: postacute care transfer at the end of the index hospitalization and ED return visit within 30 days after the index hospitalization (for those who survived to hospitalization). We used intensive care unit admission from the AGU as a safety surrogate of either strategy.

### Data

For all patients, data regarding the index hospitalization were available, as well as data concerning the year preceding and the 30 days following the index hospitalization.

### Structured data

We collected the following patient characteristics: age, sex, hospitalization in the AGU in a 12-month period before the index hospitalization (Yes/No) and hospitalization in the AGU after an ED visit in the hospital in which the ED was located (Yes/No) (inter-hospital transfers). We calculated the Charlson comorbidity index using the method validated for medico-administrative data in hospitalized patients based on ICD-10 codes [24], and these index values were divided into four classes (0, 1–2, 3–4, ≥ 5).

We also collected patients’ principal diagnoses, according to ICD10, coded during hospitalization (i.e., the ones justifying the hospitalization). The degree of severity of undernutrition was also collected and categorized as none, mild to moderate (E44.0/E.44.1) or severe (E.43.0).

Taking into account the coded diagnoses as well as the wards where the patient stayed during hospitalization, each hospital stay was assigned to a diagnosis-related group (DRG) [25]. For example, heart failure in a patient without another medical condition will have the same coding diagnosis (heart failure) but will not have the same coding DRG according to the level of severity such as heart failure complicated by cardiogenic shock in a patient with diabetes. Based on the DRG, we identified the degree of severity to which the patient belonged, ranging from 1 to 4.

Hospital LOS was divided into AGU LOS and time to AGU admission. The time to AGU admission was defined as the time between ED arrival and AGU admission in days.

### Unstructured data

Living conditions (home/institution), the presence of home helpers (nurse, nursing assistant, and physiotherapist), autonomy (normal/dependent for at least one activity of daily living [ADL]/dependent for all ADLs) and cognitive disorders (none/mild/moderate/severe) were identified by text mining from hospitalization reports. Cognitive disorders were considered to be mild when the Mini Mental Status Examination (MMSE) [26] was between 20 and 30, moderate between 10 and 19 and severe < 10. We used a simple keyword and/or regular expression matching approach [27].

Clinical signs of life-threatening conditions were identified by text mining from ED visit reports. Details of the clinical signs collected by text mining are available in Appendix 1.

### Statistical analysis

#### Descriptive analysis

We described patient characteristics as frequencies (percentages) for categorical variables and means (standard deviation) or medians (interquartile range, IQR) for continuous variables. We used an inverse probability of treatment weighting (IPTW) approach to balance the differences in baseline variables between the two strategies [28]. The study sample characteristics before and after imputation as well as before and after IPTW were described.

#### Missing data management

The numbers and percentages of missing data by variables are available in Appendix 2A. We used multiple imputation [29] to account for missing data in patient characteristics under the assumption of missingness at random (MAR) [29]. Analyses were conducted on 30 imputed datasets created using multiple imputation by chained equations, and all estimates were obtained by combining the results from the imputed datasets, applying Rubin's rules [30].

#### Propensity score

We used a multivariable logistic regression model to estimate the probability of a patient being admitted directly to the AGU given their baseline characteristics (corresponding to the propensity score). Variables considered important for prognosis or that may confound the treatment–outcome relationship were included in the propensity score model as follows: age, sex, Charlson comorbidity index, hospitalization in an AGU in a 12-month period before the index hospitalization, living conditions, cognitive disorders, autonomy, and nutritional status. Principal diagnosis and the DRG degree of severity were also included in the model. Standardized differences were calculated to assess the balanced distribution of patient characteristics across intervention groups, with a threshold of 10% designated to indicate clinically meaningful imbalance [23].

#### Regression models

For each outcome, an IPTW-weighted univariate linear or logistic regression model was built to estimate the effect of DA on each outcome’s probability.

#### Sensitivity analyses

We performed several sensitivity analyses to evaluate the consistency of the models. In the first analysis, we estimated the propensity score without principal diagnosis and/or the DRG degree of severity. In the second one, we analysed only patients with a full dataset, without multiple imputation. We performed these sensitivity analyses because principal diagnosis and degrees of severity were collected based on diagnostic codes and DRG, which were completed at the end of hospitalization. Finally, we performed a sensitivity analysis without exclusion based on diagnosis and positivity assumption and in which diagnosis were classified into several large categories.

Statistical analyses were conducted using R statistical software version 3.6.3 (R Foundation for Statistical Computing, Vienna, Austria). Data management was performed on the data warehouse with the sparklyr package [31] and multiple imputations were carried out with the mice package [32].

## Results

### Patient characteristics

**Fig. 1:**
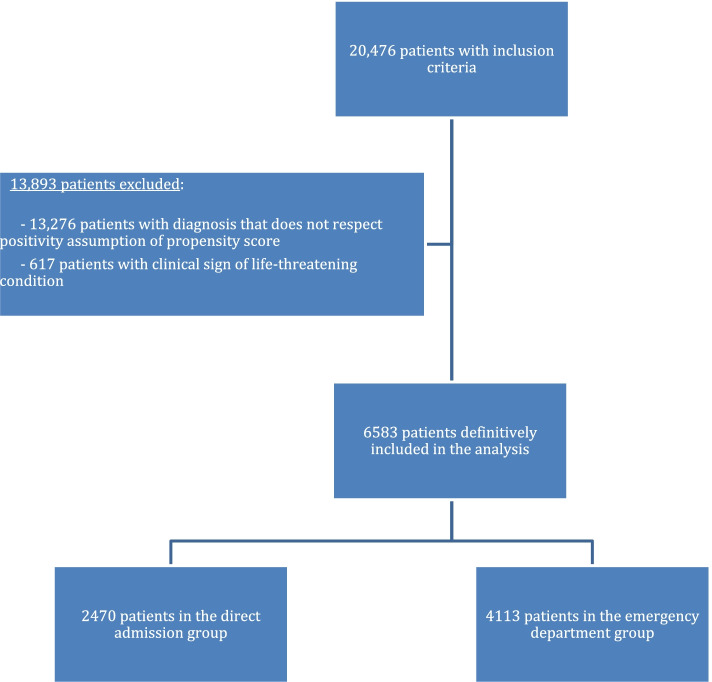
Study Flow Chart

Among the 20,416 patients admitted to an AGU during the study period, 6583 were included in the study: 37,5% (*n* = 2470) in the DA group and 62,5% (*n* = 4113) in the ED group (Table 1). The Study Flow chart is shown in Fig. 1.

**Table 1:** Caracteristics of study population

	**Admission after ED visit**	**Direct admission**	**Total**
	4113 (62.5%)	2470 (37.5%)	6583
**Age m(± SD)**	89.3 (± 6.0)	89.3 (± 5.9)	89.3 (± 5.9)
**Sex**			
Men	1352 (32.9%)	798 (32.3%)	2150 (32.7%)
Women	2761 (67.1%)	1672 (67.7%)	4433 (67.3%)
**Charlson comorbidity index**			
0	396 (9.6%)	232 (9.4%)	628 (9.5%)
1 to 2	1735 (42.2%)	1036 (42.0%)	2771 (42.1%)
3 to 4	1256 (30.5%)	752 (30.5%)	2009 (30.5%)
≥ 5	726 (17.7%)	449 (18.2%)	1175 (17.8%)
**Hospitalization in the previous year**			
No	2373 (57.7%)	1334 (54.0%)	3707 (56.3%)
Yes	1740 (42.3%)	1136 (46.0%)	2876 (43.7%)
**Undernutrition**			
None	1695 (41.2%)	1017 (41.2%)	2712 (41.2%)
Mild to moderate	1226 (29.8%)	735 (29.8%)	1961 (29.8%)
Severe	1193 (29.0%)	718 (29.1%)	1910 (29.0%)
**Living conditions**			
Home	3622 (88.1%)	2179 (88.2%)	5800 (88.1%)
Institution	491 (11.9%)	291 (11.8%)	783 (11.9%)
**Cognitive disorders**			
None	1034 (25.1%)	625 (25.3%)	1659 (25.2%)
Mild	1009 (24.5%)	597 (24.2%)	1606 (24.4%)
Moderate	1388 (33.8%)	856 (34.6%)	2244 (34.1%)
Severe	682 (16.6%)	392 (15.9%)	1074 (16.3%)
**Autonomy**			
Normal	560 (13.6%)	320 (13.0%)	880 (13.4%)
Dependent for at least one ADL	3261 (79.3%)	1971 (79.8%)	5232 (79.5%)
Dependent for all ADL	292 (7.1%)	179 (7.2%)	471 (7.1%)
**Presence of home helpers**			
No	2220 (54%)	1304 (52.8%)	3525 (53.5%)
Yes	1893 (46%)	1166 (47.2%)	3058 (46.5%)
**Principal diagnoses**			
Dementia and/or confusion	1286 (31.3%)	763 (30.9%)	2049 (31.1%)
Heart failure	712 (17.3%)	443 (17.9%)	1155 (17.5%)
Rheumatologic diagnoses	478 (11.6%)	281 (11.4%)	759 (11.5%)
Hematologic diagnoses (excluding oncology)	409 (9.9%)	247 (10.0%)	656 (10.0%)
Oncologic diagnoses	343 (8.3%)	207 (8.4%)	550 (8.4%)
Acute renal failure	332 (8.1%)	200 (8.1%)	532 (8.1%)
Endocrinologic and nutritional diagnoses	279 (6.8%)	166 (6.7%)	446 (6.8%)
Problems related to living conditions	145 (3.5%)	87 (3.5%)	232 (3.5%)
Dermatologic diagnoses	93 (2.3%)	55 (2.2%)	148 (2.2%)
Chronic renal failure	36 (0.9%)	21 (0.9%)	57 (0.9%)
**Degree of severity**			
1	193 (4.7%)	118 (4.8%)	310 (4.7%)
2	571 (13.9%)	340 (13.8%)	910 (13.8%)
3	2602 (63.3%)	1556 (63.0%)	4158 (63.2%)
4	748 (18.2%)	457 (18.5%)	1205 (18.3%)

*Note*: due to weightings, counts have been rounded to the nearest integer

Approximately half were older than 90 years, and 67% were women (*n* = 4433). The vast majority of patients were living at home (88,1%; *n* = 5800), 13,4% (*n* = 880) were independent for ADLs, and 46,5% (*n* = 3078) were receiving help at home (nursing and/or physiotherapy). The most common diagnoses were dementia and/or confusion (31,3%), and the most common severity degree was 3 out of 4 (63,2%) (Table 1). Among all patients, 43.7% had at least one hospitalization in AGU in the previous year: 42.3% in the ED group vs. 46.0% in the DA group. Among those admitted after an ED visit, 43,5% (*n* = 1788) were admitted to the AGU after an inter-hospital transfer. Less than 1% of patients had been admitted to an intensive care unit during their hospitalization.

The study population characteristics before multiple imputations and IPTW, as well as after multiple imputation and before IPTW, are available in Appendix 2A and B.

### Propensity scores

Propensity scores ranged from 0.112 to 0.948 in the DA group and from 0.109 to 0.882 in the ED group. Positivity assumptions were assessed graphically by plotting the distribution of the propensity score in both intervention groups (Appendix 3). After IPTW using stabilized weights was applied, all 10 covariates in the planned propensity score had weighted standardized differences below 10%.

### Outcomes

The median hospital LOS was 11.8 days (Q1-Q3 = 7.0–17.2) in the DA group and 13.0 days (Q1-Q3 = 8.8–18.4) in the ED group. The median AGU LOS was 11.8 days (Q1-Q3 = 7.0–17.2) and 11.9 days (Q1-Q3 = 7.8–17.0) in the DA group and in the ED group, respectively. The median time to AGU admission was 0.9 (Q1-Q3 = 0.7–1.2).

ED return visits in the month following hospitalization occurred in 3,7% (*n* = 241) of patients: 3,5% (*n* = 81) in the DA group and 4,3% (*n* = 160) in the ED group.

Transfer in postacute care at the end of hospitalization occurred in 48,8% (*n* = 2975) of patients: 46,6% (*n* = 1086) in the DA group and 50,2% *(n* = 1889) in the ED group. The results of the univariate logistic regression are summarized in Table 2. DA was associated with a shorter LOS in all patients (estimate = -1.28; 95% CI = -1.76—-0.80) as well as after exclusion of those who died during hospitalization (estimate = -1.38; 95% CI = -1.86—-0.89). It was not associated with a significant risk of transfer to an intensive care unit. In terms of morbidity, DA was also associated with a lower likelihood of postacute care transfer (OR = 0.87; 95% CI = 0.77–0.97) for those who survived to hospitalization, but not with a statistically different risk of readmission to the ED within 30 days (OR (IC)). The results from all sensitivity analyses were similar for LOS, postacute care transfer and ED return visit in the month following hospitalization at the exception of ED return visit which was significantly lower in the DA group when the analysis was performed without exclusion based on diagnosis and positivity assumption and in which diagnosis were classified into several large categories (Appendix 4).

**Table 2:** Results from regression models

	**Hospital LOS**^a^				**ED return visit**^b^		**Postacute care transfer**^b^		**Transfer in intensive care unit**^b^	
	**All patients**		**Survivors only**							
	**estimate**	**95%CI**	**estimate**	**95%CI**	**OR**	**95%CI**	**OR**	**95%CI**	**OR**	**95%CI**
**Direct admission**										
No	Ref		Ref		Ref		Ref		Ref	
Yes	-1,28	-1,76—-0.80	-1,38	-1,86—-0.89	0.80	0.60—1.08	0.87	0.77 – 0.97	0.48	0.09—2.53

^a^Results were estimated from linear regression model

^b^Results were estimated from logistic regression model

## Discussion

In this study, we report that DA to an AGU, compared with admission after an ED visit, was associated with shorter hospital LOS. On the other hand, a lower likelihood of postacute care transfer was found in this group, but no significant association with ED return visit within 30 days, two outcomes used as a proxies for morbidity. The strengths of this paper include a large sample size with data from 19 different AGUs in Paris and the limited previous literature on the subject.

The study population that allowed us to report these results is representative of the elderly hospitalized in France. Approximately 60% of patients were undernourished. This number is similar to that found in the available literature, as the percentages in hospitalized patients range between 30 and 70% according to the diagnostic criteria used [33]. Approximately 15% were living in institutions, which is approximately the same percentage as in the general population (people over 75 years) [34]. However, more than 70% had a cognitive disorder, and approximately 80% were dependent on at least one ADL, which is higher than that in the general population [35, 36] but not surprising given the vulnerability of patients admitted to AGU.

With regard to the hospitalization of elderly people for acute health problems, the effectiveness of hospitalization in a geriatric care unit is well established. Several studies have shown that compared to conventional hospitalization, admission of an elderly patient to an AGU was associated with better outcomes [15–17] and a shorter hospital LOS [17].

We show that AGU LOS was quite similar in the DA and ED groups, and it appears that the difference in hospital LOS was largely due to the time to AGU admission. Only two studies assessed direct admission (DA) to an AGU [18, 19] compared to post ED admission. They showed contrasting results. One monocentric study reported that patients admitted after an ED visit experienced more complications during hospitalization (such as acute urine retention) and had a lower frequency of returning home [19]. In another study, the authors did not report any differences between the two strategies in terms of LOS, mortality or discharge dispositions in a population of nursing home residents [18]. In these studies, the time to AGU admission was not reported.

However, this is an important point where we do know, through various studies that have been conducted [37, 38] that ED waiting time and overcrowding are associated with higher morbidity. For example, elderly patients are likely to experience delirium and/or adverse events related to an extended ED length of stay [39, 40]. Moreover, regardless of the morbidity issue, shortening this time in the ED could reduce associated health-care costs [41]. These two major considerations appear sufficient to recommend DAs as often as possible. Despite this, one could imagine that the absence of an initial assessment by an emergency physician might cause an underestimation of the initial severity and a greater proportion of early transfer to the intensive care unit [42]. Therefore, we considered transfer to an intensive care unit as a safety criterion of the DA strategy. Our results showed both a low probability of transfer to the intensive care unit (less than 1%) and no difference between groups. This finding suggests that the initial decision of the doctor to plan a DA for the patient, and therefore the absence of an assessment by an emergency physician, is a relatively safe strategy and is not putting the patient at risk of improper referral and retransfer to intensive care.

In this paper, we chose postacute care transfer at the end of the index hospitalization and ED return visit within 30 days as proxies for morbidity on the assumption that extended ED length of stay might impact elderly patients’ health not only throughout the current hospitalization but also during the following months, especially when considering the frailty of these patients. The association between frailty and adverse outcomes, such as falls, disability, hospitalization, care home admission and mortality, has been largely described [43–45]. Even if AGU LOS was quite similar in both groups, we found a higher risk of transfer in postacute care in the ED group than in the DA group, suggesting that negative outcomes related to extended ED stays before AGU admission might be involved in the failure of returning home and thus, higher need for postacute care transfers at the end of hospitalization. Regarding ED return visits, it is known that elderly individuals are at risk of repeated hospitalizations, which can lead to increased morbidity and health care costs. This is why reducing the repeated hospitalization rate should be a priority for national health plans.

Early unplanned ED return visits appear to be a negative marker of health care quality [37, 46]. These readmissions may result from premature hospital discharge, inadequate preparation of the patient and their family for discharge, and poor care transitions [38]. In this study, the rate of ED return visits was relatively low with no statistically significant difference between groups (3.5% in the DA group vs. 4.3% in the ED group), but at the cost of postacute care transfers.

If our results clearly support DAs to AGUs, a question arises regarding the feasibility of such an organization in hospitals with problems related to access blocks, ED overcrowdinghigh hospital occupancy [39, 40] and, more broadly, difficulties in managing patient flow over the entire geriatric pathway (acute care and postacute care) [47–49]. Increasing the number of AGU beds as well as strategies for reserving beds dedicated to DAs should be discussed to encourage physicians to reorganize around the DA pathways Some studies have shown that better management of inpatient beds is associated with increased systemic capacity and reduces the number of ED access blocks [7, 50]. Thus, the creation of beds dedicated to DAs can only be achieved if the total numbers of geriatric beds is increased, including in long-term care facilities.

### Limitations

Our study has several limitations. First, the choice of strategy (DA vs. ED) was not randomly assigned, and potential confounding by indication could bias our analyses. This concern was at least partially controlled by IPTW weighting based on a propensity score showing balanced baseline characteristics between groups, although unmeasured confounding can never be ruled out. Second, our propensity score included two variables based on the diagnosis code and DRG: principal diagnosis and degree of severity. This coding is completed at the end of hospitalization and could lead to errors or variations such as under- or upcoding [51–56]. However, the results from all sensitivity analyses were similar to our main analysis, which supports the robustness of our results. The fact that the ED return visit is significantly lower in the DA group when the analysis is performed without exclusion based on positivity assumption reinforces the interest of using the propensity score. Third, follow-up data only concern those available in the health data warehouse of APHP. For example, if a patient is readmitted in the ED in a hospital other than those involved in the health data warehouse of APHP, the information will not be available. This issue can cause an underestimation of the number of ED visits, yet we can find no reason why this may concern one group more than the other and hence bias our results. Fourth, we did not have information on access to primary care or insurance status while it seems these could have been interesting to include in the propensity score model, especially given that DA is organized by a patient’s GP. However, it is known that in France, only 3 million of persons (5.5% of the French population) do not have a reference doctor, probably even less among elderly patients; therefore, we can assume that this variable would not have interfered in the calculation of the score [7]. The same applies to insurance status. French state health insurance, also called social security, does not cover all medical costs, and the reimbursement rate varies depending on the type of care. To supplement the reimbursements paid by social security, supplementary health insurance can be obtained. Among retired people, approximately 95% are covered by supplementary health insurance [8].

Finally, some variables were identified by text mining from hospitalization reports. In the medical field, text mining approaches encounter many obstacles, such as grammar mistakes, different writing forms of medical terms and ambiguity in abbreviation terms [57]. All of these issues might interfere with term recognition and lead to an underestimation of the prevalence of the variable sought. However, medical reports were completed the same way for all patients, and there was no reason that would have led to a bias between groups.

## Conclusion

Direct admission is associated with shorter hospital LOS and fewer postacute care transfers. No significant association with readmission to the ED within one month following AGU hospitalization or with ICU transfers was found. DA to the AGU should be prioritized, and reorganization of the geriatric pathway around the DA should be encouraged due to the frailty of elderly individuals.

## Supplementary Information

**Additional file 1.**

## Data Availability

Data supporting this study can be made available on request (claire.hassen-khodja@aphp.fr), on condition that the research project is accepted by Scientific and Ethical Committee of Assistance Publique – Hopitaux de Paris (AP-HP) clinical data warehouse.
