# Supplementary material for: The effect of direct admission to acute geriatric units compared to admission after an emergency department visit on length of stay, postacute care transfers and ED return visits
Source: BMC Geriatr. 2022 Jul 4;22:555. doi: 10.1186/s12877-022-03241-x (PMC9254499; doi:10.1186/s12877-022-03241-x)
Supplement: Supplementary file 1 — Additional file 1. [file 12877_2022_3241_MOESM1_ESM.docx]

**Appendix 1. Exclusions based on medical conditions criteria**

Clinical signs of life threatening conditions

Clinical signs of life threatening conditions were identified by text mining on the ED medical records. ED medical records consist of some administrative information as well as medical observations written by the medical team in charge of the patient. The absence or presence of clinical signs of life threatening conditions are routinely reported. The following words and expression were collected by text mining:

- mostling and/or skin recoloration time > 3 seconds and/or extended skin recoloration time as well as « signs of shock » or « state of shock »
- fluid administration
- respiratory distress and/or respiratory retractions and/or thoraco-abominal asynchrony
- cyanosis
- sweating

Living conditions, autonomy and cognitive disorders

In AGU, hospitalization reports are standardized and always structured in the same way. At the beginning of the report, a lifestyle section must be included and specify the patient’s degree of autonomy, cognitive assessment, and living conditions. MMSE is collected routinely except when it is impossible to perform (due to severe dementia). In this case, it is specified in the report and the patients are reclassified as severe dementia.

For living conditions, the following terms were searched: home, apartment, house, villa, institution and nursing home.

For home helpers, the following terms were searched: nurse, caregiver, carer, housekeeper, physiotherapist

For autonomy, the following terms were searched: autonomous, dependent, bedridden, cane, armchair, walker as well as the ADL score.

For cognitive disorders, the terms MMS and MMSE were searched.

Medical conditions excluded because of no respect of the positivity assumption of propensity score

Propensity score methods assume that each patient has both positive probability of receiving intervention and receiving control – called the positive assumption of propensity score.

In our study, for some diagnoses, the positive assumption of propensity score was not respected. It means that when this diagnosis is suspected, the patient is always referred to ED or always admitted directly. Thus, we excluded all diagnoses that did not respect the positive assumption of propensity score:

| **ICD-10 CHAPTERS** | | | **Excluded** | **Included** |
| --- | --- | --- | --- | --- |
| I | A00–B99 | Certain infectious and parasitic diseases | Excluded except A46 | A46 classified with the chapter XII |
| II | C00–D48 | Neoplasms |  | Yes |
| III | D50–D89 | Diseases of the blood and blood-forming organs and certain disorders involving the immune mechanism |  | Yes |
| IV | E00–E90 | Endocrine, nutritional and metabolic diseases |  | Yes |
| V | F00–F99 | Mental and behavioural disorders | Excluded except F68 and F69 | F68 and F69 classified as « Dementia and/or confusion » |
| VI | G00–G99 | Diseases of the nervous system | Yes |  |
| VII | H00–H59 | Diseases of the eye and adnexa | Yes |  |
| VIII | H60–H95 | Diseases of the ear and mastoid process | Yes |  |
| IX | I00–I99 | Diseases of the circulatory system | Excluded except I50 | I50 (Heart Failure) |
| X | J00–J99 | Diseases of the respiratory system | Yes |  |
| XI | K00–K93 | Diseases of the digestive system | Yes |  |
| XII | L00–L99 | Diseases of the skin and subcutaneous tissue |  | Yes |
| XIII | M00–M99 | Diseases of the musculoskeletal system and connective tissue |  | Yes |
| XIV | N00–N99 | Diseases of the genitourinary system | Excluded except N0, N10, N12, N13, N17, N18 and N19 | N0, N12, N13, N18 and N19 who were classified as « Chronic renal failure » as well as N10 and N17 who were classified as « Acute renal dysfonction » |
| XV | O00–O99 | Pregnancy, childbirth and the puerperium |  |  |
| XVI | P00–P96 | Certain conditions originating in the perinatal period |  |  |
| XVII | Q00–Q99 | Congenital malformations, deformations and chromosomal abnormalities |  |  |
| XVIII | R00–R99 | Symptoms, signs and abnormal clinical and laboratory findings, not elsewhere classified | Excluded except R33 | R33 who was classified as « Acute renal failure » |
| XIX | S00–T98 | Injury, poisoning and certain other consequences of external causes | Yes |  |
| XX | V01–Y98 | External causes of morbidity and mortality | Yes |  |
| XXI | Z00–Z99 | Factors influencing health status and contact with health services | Excluded except Z59, Z60, Z63, Z72, Z74 and Z75 | Z59, Z60, Z63, Z72, Z74 and Z75 who were classified as « Problems related to living conditions » |

**Appendix 2A - Characteristics of study population before multiple imputation and IPTW**

|  | **Admission after ED visit** | **Direct admission** | **Total** |
| --- | --- | --- | --- |
|  | 4113 (62.5 %) | 2470 (37.5 %) | 6583 |
| **Age** | 89.5 (± 6.0) | 88.9 (± 5.8) | 89.3 (± 5.9) |
| **Sex** |  |  |  |
| Men | 1384 (33.6 %) | 783 (31.7 %) | 2167 (32.9 %) |
| Women | 2729 (66.4 %) | 1687 (68.3 %) | 4416 (67.1 %) |
| **Charlson comorbidity index** |  |  |  |
| 0 | 409 (9.9 %) | 927 (9.2 %) | 636 (9.7 %) |
| 1 to 2 | 1735 (42.2 %) | 1034 (41.9 %) | 2769 (42.1 %) |
| 3 to 4 | 1260 (30.6 %) | 760 (30.8 %) | 2020 (30.7 %) |
| ≥ 5 | 709 (17.2 %) | 449 (18.2 %) | 1158 (17.6 %) |
| **Hospitalization in the previous year** |  |  |  |
| No | 2438 (59.3 %) | 1233 (49.9 %) | 3671 (55.8 %) |
| Yes | 1675 (40.7 %) | 1237 (50.1 %) | 2912 (44.2 %) |
| **Undernutrition** |  |  |  |
| None | 1648 (40.1 %) | 1080 (43.7 %) | 2728 (41.4 %) |
| Mild to moderate | 1244 (30.2 %) | 710 (28.7 %) | 1954 (29.7 %) |
| Severe | 1221 (29.7 %) | 680 (27.5 %) | 1901 (28.9 %) |
| **Living conditions** |  |  |  |
| Home | 3422 (83.2 %) | 1950 (78.9 %) | 5372 (81.6 %) |
| Institution | 365 (8.9 %) | 345 (14.0 %) | 710 (10.8 %) |
| Missing data | 326 (7.9 %) | 175 (7.1 %) | 501 (7.6 %) |
| **Cognitive disorders** |  |  |  |
| None | 533 (13.0 %) | 273 (11.1 %) | 806 (12.2 %) |
| Mild | 523 (12.7 %) | 294 (11.9 %) | 817 (12.4 %) |
| Moderate | 703 (17.1 %) | 444 (18.0 %) | 1147 (17.4 %) |
| Severe | 340 (8.3 %) | 179 (7.2 %) | 519 (7.9 %) |
| Missing data | 2014 (49.0 %) | 1280 (51.8 %) | 3294 (50.0 %) |
| **Autonomy** |  |  |  |
| Normal | 433 (10.5 %) | 270 (7.2 %) | 703 (10.7 %) |
| Dependent for at least one ADL | 2705 (65.8 %) | 1697 (68.7 %) | 4402 (66.9 %) |
| Dependent for all ADL | 227 (5.5 %) | 149 (6.0 %) | 376 (5.7 %) |
| Missing data | 748 (18.2 %) | 354 (14.3 %) | 1102 (16.7 %) |
| **Presence of home helpers** |  |  |  |
| No | 2174 (52.9 %) | 1369 (55.4 %) | 3543 (53.8 %) |
| Yes | 1939 (47.1 %) | 1101 (44.6 %) | 3040 (46.2 %) |
| **Principal diagnoses** |  |  |  |
| Dementia and/or confusion | 1312 (31.9 %) | 727 (29.4 %) | 2039 (31.0 %) |
| Heart failure | 877 (21.3 %) | 251 (10.2 %) | 1128 (17.1 %) |
| Rheumatologic diagnoses | 499 (12.1 %) | 264 (10.7 %) | 763 (11.6 %) |
| Hematologic diagnoses (excluding oncology) | 312 (7.6 %) | 376 (15.2 %) | 688 (10.5 %) |
| Oncologic diagnoses | 222 (5.4 %) | 336 (13.6 %) | 558 (8.5 %) |
| Acute renal failure | 404 (9.8 %) | 122 (4.9 %) | 526 (8.0 %) |
| Endocrinologic and nutritional diagnoses | 211 (5.1 %) | 229 (9.3 %) | 440 (6.7 %) |
| Problems related to living conditions | 162 (3.9 %) | 69 (2.8 %) | 231 (3.5 %) |
| Dermatologic diagnoses | 75 (1.8 %) | 77 (3.1 %) | 152 (2.3 %) |
| Chronic renal failure | 39 (0.9 %) | 19 (0.8 %) | 58 (0.9 %) |
| **Degree of severity** |  |  |  |
| 1 | 133 (3.2 %) | 184 (7.5 %) | 317 (4.8 %) |
| 2 | 506 (12.3 %) | 429 (17.4 %) | 935 (14.2 %) |
| 3 | 2611 (63.5 %) | 1494 (60.5 %) | 4105 (62.4 %) |
| 4 | 812 (19.7 %) | 354 (14.3 %) | 1166 (17.7 %) |
| Missing data | 51 (1.2) | 9 (0.4 %) | 60 (0.9 %) |
|  |  |  |  |
|  | | | |
| Note: Imputation is the process of replacing missing data with substituted values. Multiple imputation is an imputation process where the imputed values are drawn *m* times from a distribution. At the end of this step, there should be *m* completed datasets. | | | |

**Appendix 2B - Caracteristics of study population after multiple imputation and before IPTW**

|  | **Admission after ED visit** | **Direct admission** | **Total** |
| --- | --- | --- | --- |
|  | 4113 (62.5 %) | 2470 (37.5 %) | 6583 |
| **Age m(± SD)** | 89.5 (± 6.0) | 88.9 (± 5.8) | 89.3 (± 5.9) |
| **Sex** |  |  |  |
| Men | 1384 (33.6 %) | 783 (31.7 %) | 2167 (32.9 %) |
| Women | 2729 (66.4 %) | 1687 (68.3 %) | 4416 (67.1 %) |
| **Charlson comorbidity index** |  |  |  |
| 0 | 409 (9.9 %) | 227 (9.2 %) | 636 (9.7 %) |
| 1 to 2 | 1735 (42.2 %) | 1034 (41.9 %) | 2769 (42.1 %) |
| 3 to 4 | 1260 (30.6 %) | 760 (30.8 %) | 2020 (30.7 %) |
| ≥ 5 | 709 (17.2 %) | 449 (18.2 %) | 1158 (17.6 %) |
| **Hospitalization in the previous year** |  |  |  |
| No | 2438 (59.3 %) | 1233 (49.9 %) | 3671 (55.8 %) |
| Yes | 1675 (40.7 %) | 1237 (50.1 %) | 2912 (44.2 %) |
| **Undernutrition** |  |  |  |
| None | 1648 (40.1 %) | 1080 (43.7 %) | 2728 (41.4 %) |
| Mild to moderate | 1244 (30.2 %) | 710 (28.7 %) | 1954 (29.7 %) |
| Severe | 1221 (29.7 %) | 680 (27.5 %) | 1901 (28.9 %) |
| **Living conditions** |  |  |  |
| Home | 3703 (90.0 %) | 2093 (84.7 %) | 5796 (88.1 %) |
| Institution | 410 (10.0 %) | 377 (15.3 %) | 787 (11.9 %) |
| **Cognitive disorders** |  |  |  |
| None | 1076 (26.2 %) | 579 (23.4 %) | 1655 (25.1 %) |
| Mild | 1009 (24.5 %) | 604 (24.4 %) | 1612 (24.5 %) |
| Moderate | 1341 (32.6 %) | 913 (36. 9 %) | 2254 (34.2 %) |
| Severe | 687 (16.7 %) | 375 (15.2 %) | 1062 (16.1 %) |
| **Autonomy** |  |  |  |
| Normal | 543 (13.2 %) | 318 (12.9 %) | 861 (13.1 %) |
| Dependent for at least one ADL | 3272 (79.6 %) | 1972 (79.9 %) | 5244 (79.7 %) |
| Dependent for all ADL | 298 (7.3 %) | 179 (7.3 %) | 478 (7.3 %) |
| **Presence of home helpers** |  |  |  |
| No | 2174 (52.9 %) | 1369 (55.4 %) | 3543 (53.8 %) |
| Yes | 1939 (47.1 %) | 1101 (44.6 %) | 3040 (46.2 %) |
| **Principal diagnoses** |  |  |  |
| Dementia and/or confusion | 1312 (31.9 %) | 727 (29.4 %) | 2039 (31.0 %) |
| Heart failure | 877 (21.3 %) | 251 (10.2 %) | 1128 (17.1 %) |
| Rheumatologic diagnoses | 499 (12.1 %) | 264 (10.7 %) | 763 (11.6 %) |
| Hematologic diagnoses (excluding oncology) | 312 (7.6 %) | 376 (15.2 %) | 688 (10.5 %) |
| Oncologic diagnoses | 222 (5.4 %) | 336 (13.6 %) | 558 (8.5 %) |
| Acute renal failure | 404 (9.8 %) | 122 (4.9 %) | 526 (8.0 %) |
| Endocrinologic and nutritional diagnoses | 211 (5.1 %) | 229 (9.3 %) | 440 (6.7 %) |
| Problems related to living conditions | 162 (3.9 %) | 69 (2.8 %) | 231 (3.5 %) |
| Dermatologic diagnoses | 75 (1.8 %) | 77 (3.1 %) | 152 (2.3 %) |
| Chronic renal failure | 39 (0.9 %) | 19 (0.8 %) | 58 (0.9 %) |
| **Degree of severity** |  |  |  |
| 1 | 134 (3.3 %) | 185 (7.5 %) | 319 (4.8 %) |
| 2 | 511 (12.4 %) | 430 (17.4 %) | 942 (14.3 %) |
| 3 | 2641 (64.2 %) | 1499 (60.7 %) | 4139 (62.9 %) |
| 4 | 827 (20.1 %) | 356 (14.4 %) | 1183 (18.0 %) |
|  |  |  |  |
|  | | | |
| Note: Imputation is the process of replacing missing data with substituted values. Multiple imputation is an imputation process where the imputed values are drawn *m* times from a distribution. At the end of this step, there should be *m* completed datasets. | | | |

**Appendix 3. Propensity score**


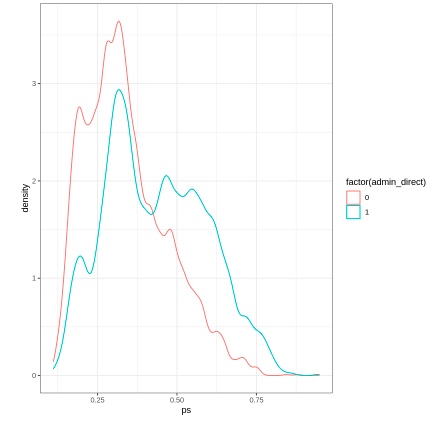


**Appendix 4A - Sensitivity analysis (propensity score without main diagnosis and level of severity)**

|  | **Hospital LOS*** | | | | **ED return visit**** | | **Transfer in post-acute care** | | **Transfer in intensive care unit**** | |
| --- | --- | --- | --- | --- | --- | --- | --- | --- | --- | --- |
|  | **All patients** | | **Survivors only** | |  |  |  |  |  |  |
|  | **estimate** | **95%CI** | **estimate** | **95%CI** | **OR** | **95%CI** | **OR** | **95%CI** | **OR** | **95%CI** |
| **Direct admission** |  |  |  |  |  |  |  |  |  |  |
| No | Ref |  | Ref |  | Ref |  |  |  | Ref |  |
| Yes | -1.69 | -2.16 - -1.22 | -1.84 | -2.32 - -1.37 | 0.78 | 0.60 - 1.08 | 0.79 | 0.71-0.87 | 0.65 | 0.13 - 3.34 |

Note

*Results were estimated from linear regression model

**Results were estimated from logistic regression model

**Appendix 4B - Sensitivity analysis (propensity score without level of severity)**

|  | **Hospital LOS*** | | | | **ED return visit**** | | **Transfer in post-acute care** | | **Transfer in intensive care unit**** | |
| --- | --- | --- | --- | --- | --- | --- | --- | --- | --- | --- |
|  | **All patients** | | **Survivors only** | |  |  |  |  |  |  |
|  | **estimate** | **95%CI** | **estimate** | **95%CI** | **OR** | **95%CI** | **OR** | **95%CI** | **OR** | **95%CI** |
| **Direct admission** |  |  |  |  |  |  |  |  |  |  |
| No | Ref |  | Ref |  | Ref |  |  |  | Ref |  |
| Yes | -1.61 | -2.08 - -1.14 | -1.69 | -2.17 - -1.21 | 0.79 | 0.59 - 1.05 | 0.84 | 0.75-0.94 | 0.50 | 0.09 - 2.65 |

Note

*Results were estimated from linear regression model

**Results were estimated from logistic regression model

**Appendix 4C - Sensitivity analysis (propensity score without main diagnosis)**

|  | **Hospital LOS*** | | | | **ED return visit**** | | **Transfer in post-acute care** | | **Transfer in intensive care unit**** | |
| --- | --- | --- | --- | --- | --- | --- | --- | --- | --- | --- |
|  | **All patients** | | **Survivors only** | |  |  |  |  |  |  |
|  | **estimate** | **95%CI** | **estimate** | **95%CI** | **OR** | **95%CI** | **OR** | **95%CI** | **OR** | **95%CI** |
| **Direct admission** |  |  |  |  |  |  |  |  |  |  |
| No | Ref |  | Ref |  | Ref |  |  |  | Ref |  |
| Yes | -1.24 | -1.72 - -0.77 | -1.42 | -1.90 - -0.94 | 0.78 | 0.59 - 1.02 | 0.82 | 0.74-0.91 | 0.57 | 0.11 - 2.96 |

Note

*Results were estimated from linear regression model

**Results were estimated from logistic regression model

**Appendix 4D - Sensitivity analysis on study population before multiple imputation**

|  | **Hospital LOS*** | | | | **ED return visit**** | | **Transfer in post-acute care** | | **Transfer in intensive care unit**** | |
| --- | --- | --- | --- | --- | --- | --- | --- | --- | --- | --- |
|  | **All patients** | | **Survivors only** | |  |  |  |  |  |  |
|  | **estimate** | **95%CI** | **estimate** | **95%CI** | **OR** | **95%CI** | **OR** | **95%CI** | **OR** | **95%CI** |
| **Direct admission** |  |  |  |  |  |  |  |  |  |  |
| No | Ref |  | Ref |  | Ref |  |  |  | Ref |  |
| Yes | -1.91 | -2.39 - -1.43 | -1.93 | -2.42 - -1.44 | 0.81 | 0.61 - 1.08 | 0.72 | 0.65-0.81 | 0.55 | 0.10 - 2.95 |

Note

*Results were estimated from linear regression model

**Results were estimated from logistic regression model

**Appendix 4E - Sensitivity analysis on study population without exclusion based on diagnosis and positivity assumption in which diagnosis were classified into several large categories**

|  | **Hospital LOS*** | | | | **ED return visit**** | | **Transfer in post-acute care** | | **Transfer in intensive care unit**** | |
| --- | --- | --- | --- | --- | --- | --- | --- | --- | --- | --- |
|  | **All patients** | | **Survivors only** | |  |  |  |  |  |  |
|  | **estimate** | **95%CI** | **estimate** | **95%CI** | **OR** | **95%CI** | **OR** | **95%CI** | **OR** | **95%CI** |
| **Direct admission** |  |  |  |  |  |  |  |  |  |  |
| No | Ref |  | Ref |  | Ref |  |  |  | Ref |  |
| Yes | -0.93 | -1.26 - -0.60 | -1.02 | -1.35 - -0.68 | 0.73 | 0.58 – 0.90 | 0.99 | 0.91 – 1.07 | 1.18 | 0.44 – 3.16 |

Note

*Results were estimated from linear regression model

**Results were estimated from logistic regression model
